# Supplementary figures and images for: Transcriptome Profile Reveals Genetic and Metabolic Mechanisms Related to Essential Fatty Acid Content of Intramuscular Longissimus thoracis in Nellore Cattle
Source: Metabolites. 2022 May 23;12(5):471. doi: 10.3390/metabo12050471 (PMC9144777; doi:10.3390/metabo12050471)

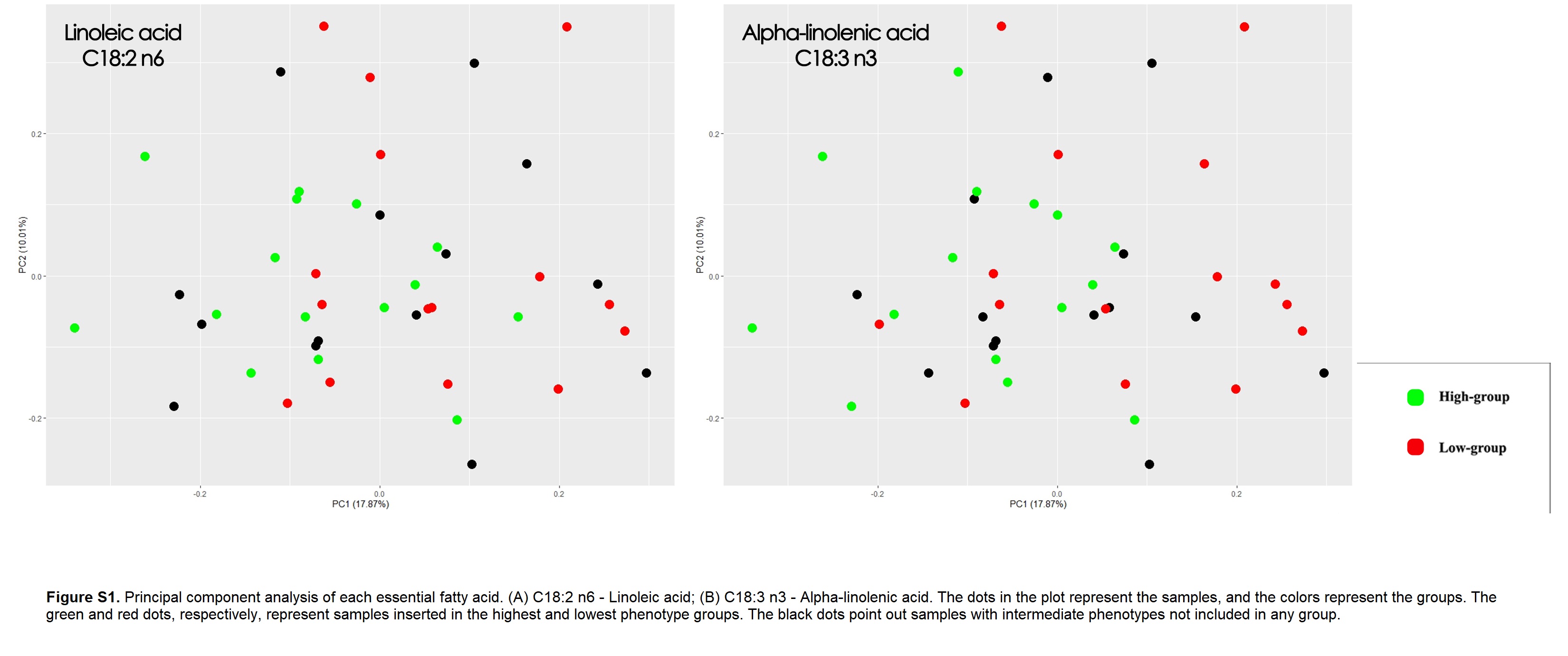

Supplement: Supplementary file 1 [file metabolites-12-00471-s001.zip › Supplementary Figure S1.jpg]

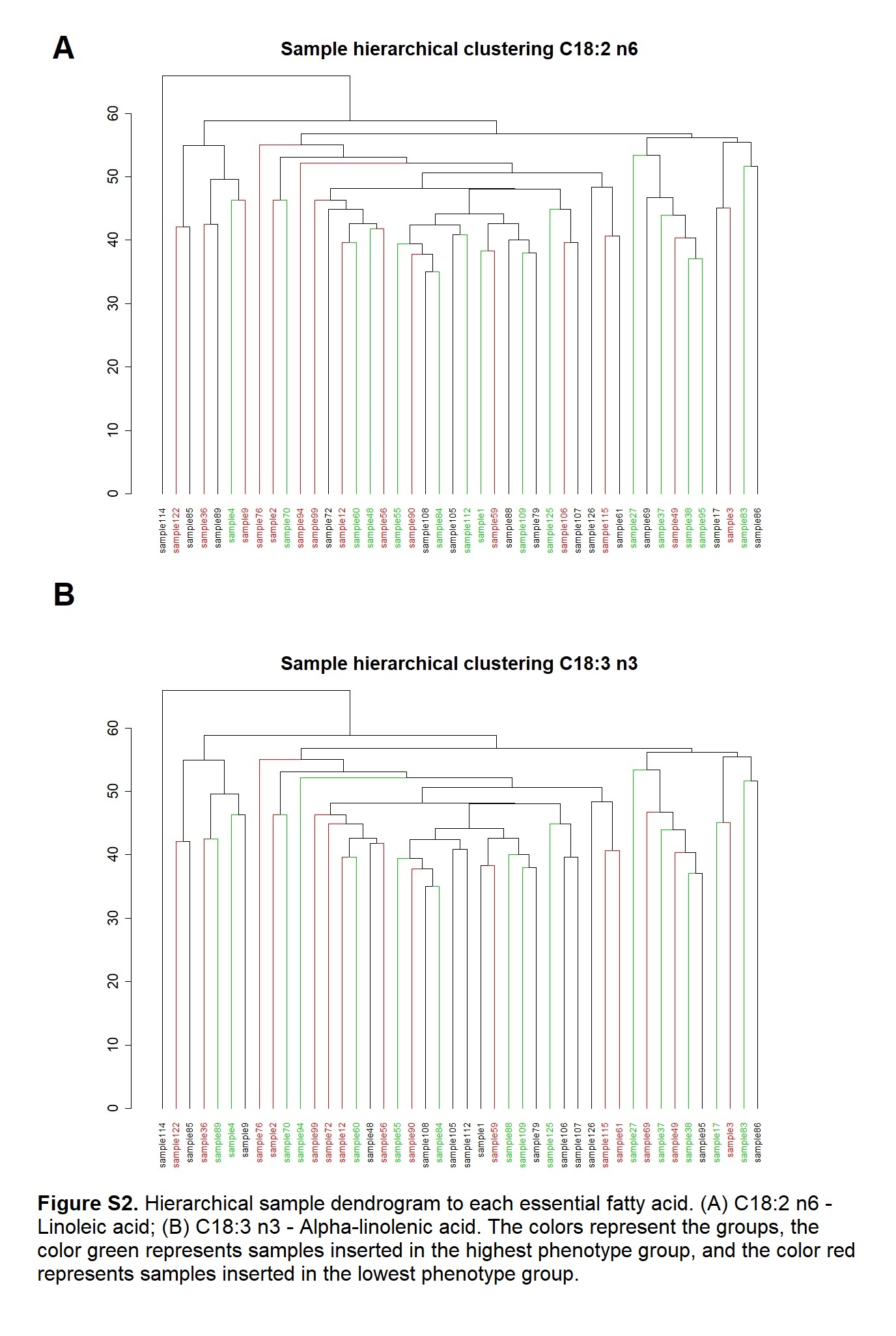

Supplement: Supplementary file 1 [file metabolites-12-00471-s001.zip › Supplementary Figure S2.jpg]

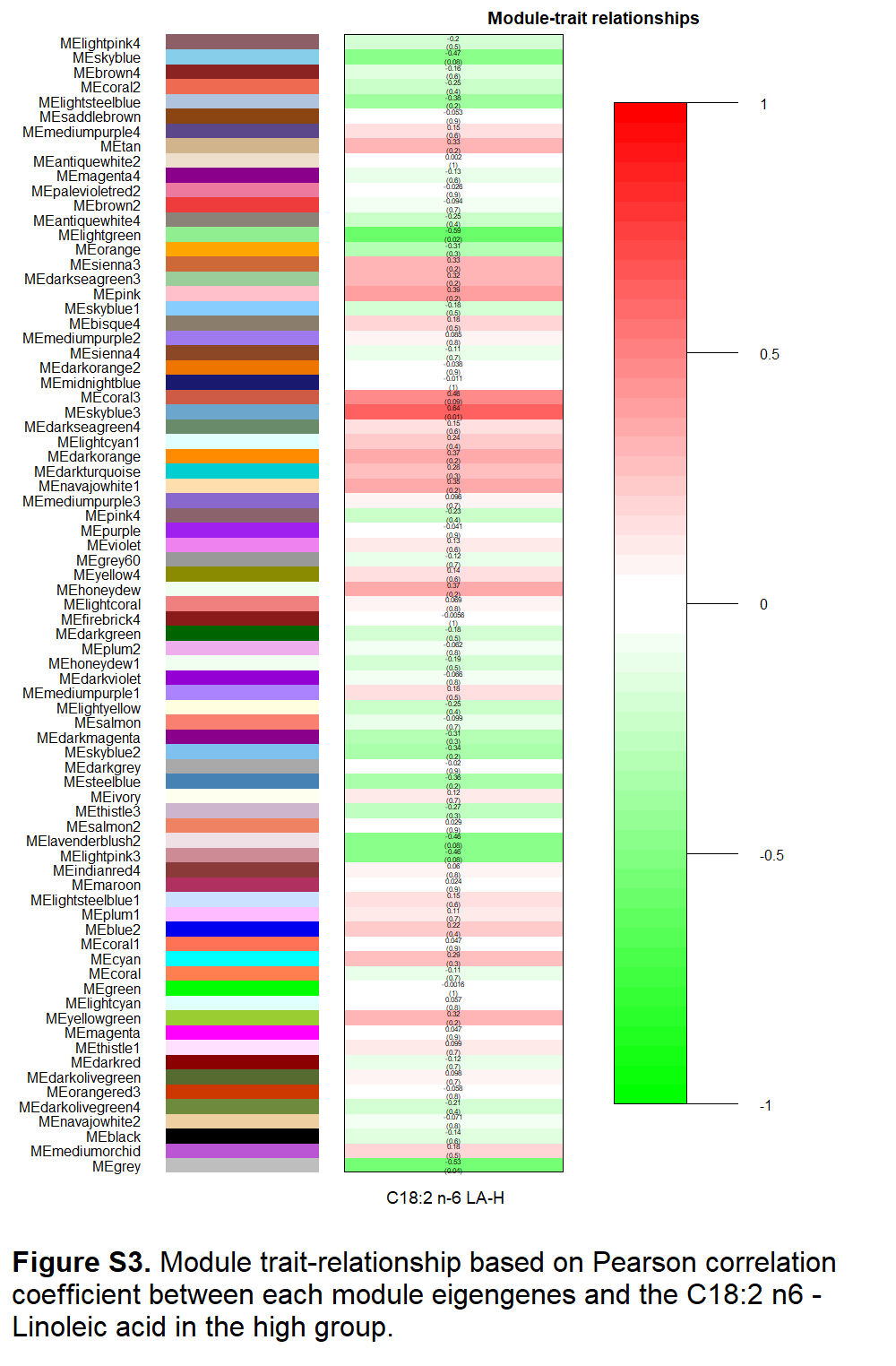

Supplement: Supplementary file 1 [file metabolites-12-00471-s001.zip › Supplementary Figure S3.png]

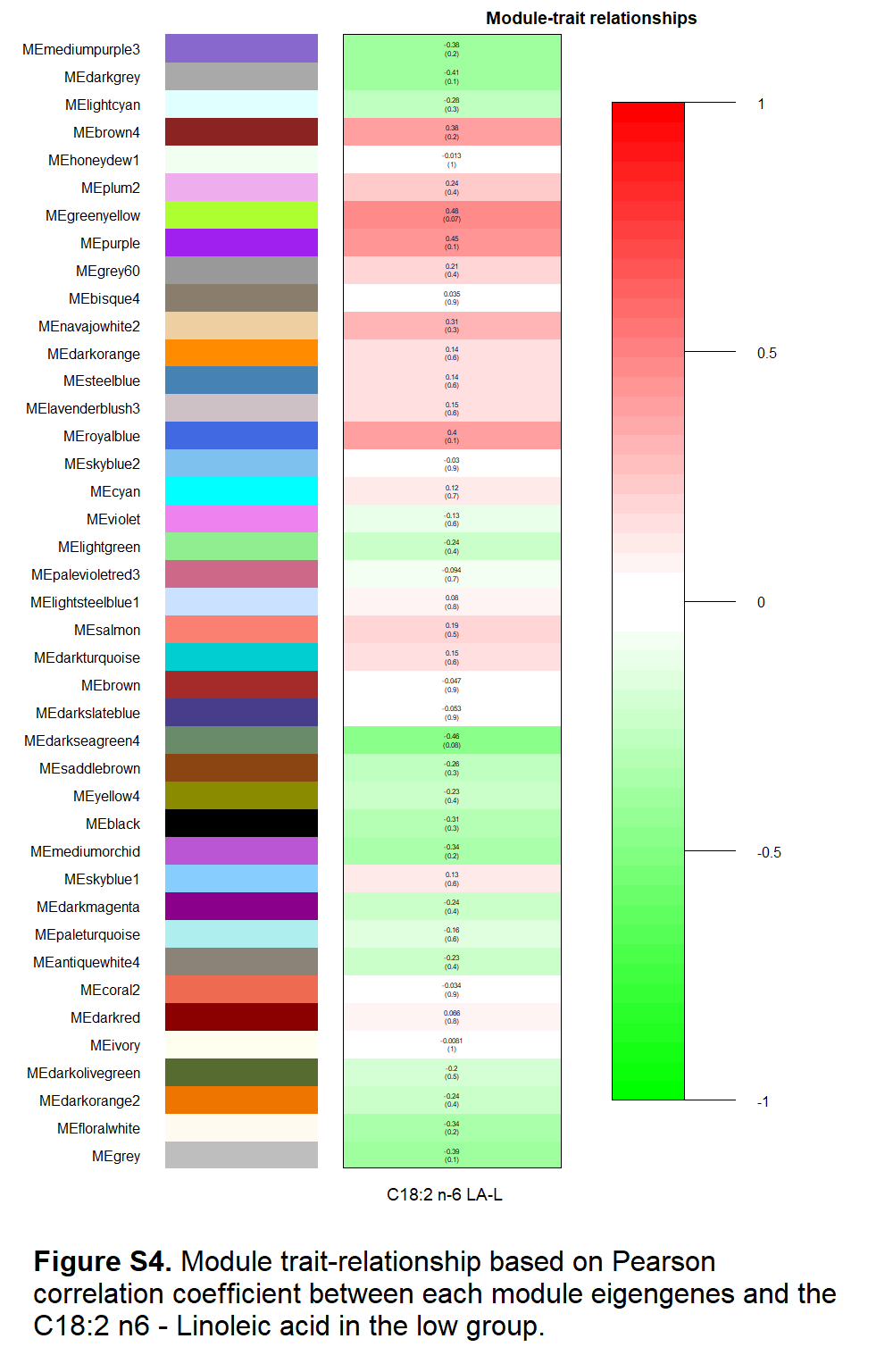

Supplement: Supplementary file 1 [file metabolites-12-00471-s001.zip › Supplementary Figure S4.png]

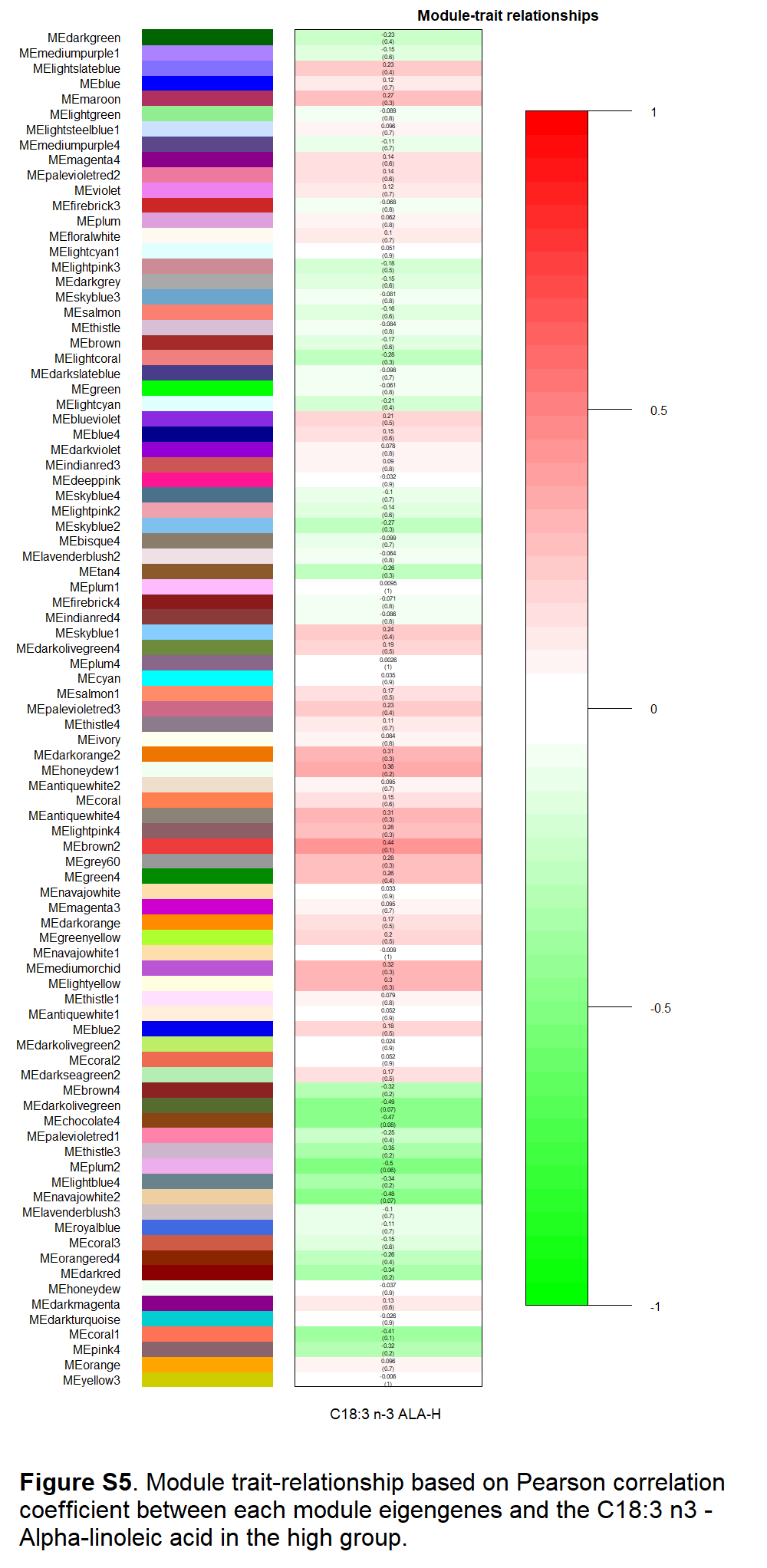

Supplement: Supplementary file 1 [file metabolites-12-00471-s001.zip › Supplementary Figure S5.png]

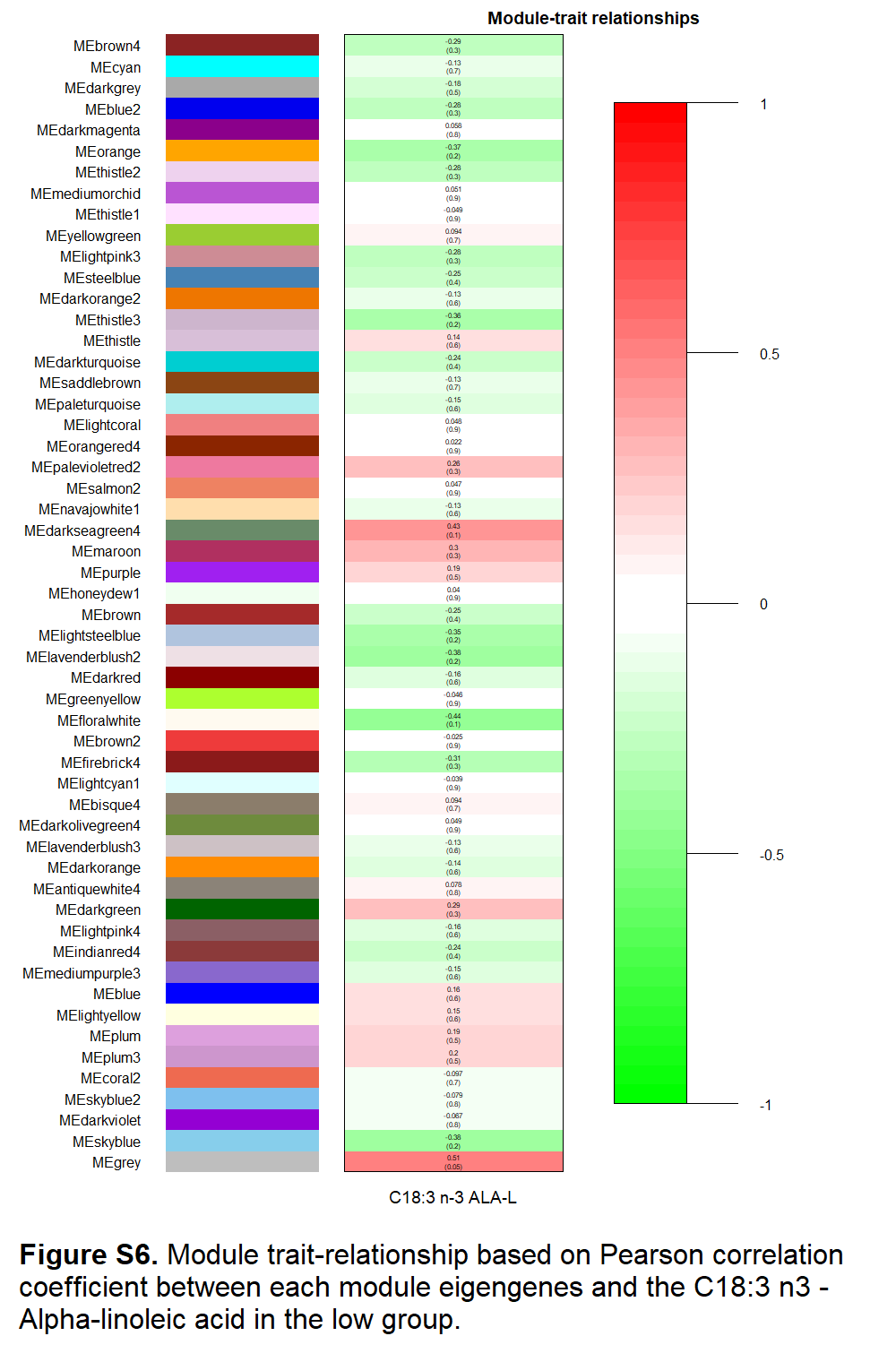

Supplement: Supplementary file 1 [file metabolites-12-00471-s001.zip › Supplementary Figure S6.png]
